# Undertaking cause-specific mortality measurement in an unregistered population: an example from Tigray Region, Ethiopia

**DOI:** 10.3402/gha.v7.25264

**Published:** 2014-09-11

**Authors:** Hagos Godefay, Atakelti Abrha, John Kinsman, Anna Myléus, Peter Byass

**Affiliations:** 1 Tigray Regional Health Bureau, Mekelle, Ethiopia; 2 WHO Collaborating Centre for Verbal Autopsy, Epidemiology and Global Health, Department of Public Health and Clinical Medicine, Umeå University, Umeå, Sweden; 3 Medical Research Council/Wits University Rural Public Health and Health Transitions Research Unit (Agincourt), School of Public Health, Faculty of Health Sciences, University of the Witwatersrand, Johannesburg, South Africa; 4 Institute of Applied Health Sciences, School of Medicine and Dentistry, University of Aberdeen, Aberdeen, United Kingdom

**Keywords:** Ethiopia, mortality, verbal autopsy, population survey, civil registration and vital statistics, evidence-based decision making

## Abstract

**Background:**

The lack of adequate documentation of deaths, and particularly their cause, is often noted in African and Asian settings, but practical solutions for addressing the problem are not always clear. Verbal autopsy methods (interviewing witnesses after a death) have developed rapidly, but there remains a lack of clarity as to how these methods can be effectively applied to large unregistered populations. This paper sets out practical details for undertaking a representative survey of cause-specific mortality in a population of several million, taking Tigray Region in Ethiopia as a prototype.

**Sampling:**

Sampling was designed around an expected level of maternal mortality ratio of 400 per 100,000 live births, which needed measuring within a 95% confidence interval of approximately ±100. Taking a stratified cluster sample within the region at the district level for logistic reasons, and allowing for a design effect of 2, this required a population of around 900,000 people, equating to six typical districts. Since the region is administered in six geographic zones, one district per zone was randomly selected.

**Implementation:**

The survey was implemented as a two-stage process: first, to trace deaths that occurred in the sampled districts within the preceding year, and second to follow them up with verbal autopsy interviews. The field work for both stages was undertaken by health extension workers, working in their normally assigned areas. Most of the work was associated with tracing the deaths, rather than undertaking the verbal autopsy interviews.

**Discussion:**

This approach to measuring cause-specific mortality in an unregistered Ethiopian population proved to be feasible and effective. Although it falls short of the ideal situation of continuous civil registration and vital statistics, a survey-based strategy of this kind may prove to be a useful intermediate step on the road towards full civil registration and vital statistics implementation.

Although a lack of adequate cause-specific mortality information at the population level is often noted and regretted, particularly in Africa and Asia, it is not always clear how the situation might be remedied in practical terms (1). While the ideal solution might be universal and compulsory death registration on a continuous basis, its implementation would require quantum shifts in terms of inter-sectoral actions and resource allocations, which are not likely to happen quickly or easily. This paper sets out a design for measuring cause-specific mortality on a population basis over a population of several million, including details of practical experience and costings.

Recent years have seen considerable methodological advances in methods for assigning cause of death in settings where death certificates are not routinely issued by physicians. These advances have centred on developments in the application of verbal autopsy, an approach involving interviewing relatives of the deceased or witnesses of a death to elicit specific items of medical history, symptoms during the final illness, and other characteristics (2). The World Health Organization (WHO) has contributed substantially to this process in releasing the WHO 2012 verbal autopsy standard (3, 4), which sets out standard items for verbal autopsy interviews and defined a set of cause of death categories covering the International Classification of Diseases version 10 (ICD-10) (5). Additionally, methods for the automated interpretation of verbal autopsy interview material have developed substantially, including the InterVA-4 model which corresponds exactly to the WHO 2012 verbal autopsy standard (6). Some validation studies for particular causes of death have been reported from research-based demographic surveillance sites, although this is not possible to do for every cause of death (7, 8). These models can also be implemented as smart-phone applications, simplifying the verbal autopsy interview process and making individual cause of death assignments immediately available (9). Thus, in terms of moving from the witnesses of a death to assigning a cause to a death, and then aggregating those causes of death over a population, there are no major technical obstacles remaining.

However, careful strategic thinking needs to be given to the logistic and methodological approaches needed to obtain meaningful measurements of cause-specific mortality from large unregistered populations, such as those of small countries or individual regions of larger countries. Populations in which physicians do not routinely issue death certificates are also generally those which do not maintain effective registration of individual citizens, and where an appreciable proportion of deaths do not occur in health facilities. Therefore, issues of identifying deaths that have occurred in a population and determining population denominators have to precede undertaking verbal autopsy interviews. Sampling from within the overall population in question to achieve both adequate and representative results is also a crucial part of the process.

The aim of this paper is to set out the detailed considerations involved in designing a cause-specific mortality survey in Tigray Region, Ethiopia, which took a specific focus on maternal and child mortality. This includes the statistical design, survey logistics, and costs involved in undertaking a representative cause-specific mortality survey among a population of approximately 5 million people.

## Study setting

Tigray Region is the northernmost of Ethiopia's nine regional states, bordered by Sudan to the west and Eritrea to the north. The population of Tigray is estimated at 5.1 million, with 82% living in rural areas. Population density varies considerably over the 50,078 km^2^ covered by the region, with a mean of 102 km^−2^. The region is subdivided into six rural zones, each of which contains a number of districts (known as *woredas*), each typically including a population of 100,000–200,000 people (10).

Recent national estimates for maternal mortality ratio from various sources led to design assumptions for maternal mortality ratio at 400 per 100,000 live births with the crude birth rate estimated at 30 per 1,000 population per year (10–12).

## Sampling strategy

The basis for sampling within the region involved assumptions about adequate numbers and the need to achieve region-wide representation, taken together with logistic considerations. Sample size considerations for cause-specific mortality surveys are complex because they depend less on total numbers of deaths and more on the number of cases for the *n*th ranked cause of death of interest, given that ranked cause-specific mortality fractions in a population tend to decline exponentially (13). Achieving regional representation requires a geographically stratified sampling strategy; in theory a fine-grained stratification (at household or village level) would be preferable. However, logistic considerations dictate a coarser stratification, as it is unrealistic in a large-scale survey process to locate a high number of randomly sampled individual households or villages across an entire region.

To make a reasonable measurement of maternal mortality, a sufficient number of pregnancy-related deaths need to be surveyed, identified from among all deaths of women of reproductive age (15–49 years). For sample size purposes, this can either be estimated as a fraction of total mortality among women of reproductive age, or in terms of the ratio of maternal deaths to live births. Here we worked with the latter; hypothetically a maternal mortality ratio (MMR) of 400 per 100,000 could be estimated within a 95% confidence interval of 300 to 522 if 54 deaths were observed out of 13,500 live births (Poisson confidence interval, using Stata 12 *cii* command). A crude birth rate of 30 per 1,000 would mean that a population base of 450,000 would yield coverage of 13,500 live births over a 1 year period. Previous work on appropriate recall periods for verbal autopsy interviews suggest that up to a 1-year recall period is adequate to avoid undue recall bias (14), and is also operationally convenient, since it involves asking people to recall deaths that have occurred in the 1-year period preceding a survey.

In this survey, to optimise logistic efficiency, deaths among women of reproductive age and deaths of children under 5 years of age were designed to be tracked in the same field operation. Maternal deaths are much less common events than under-five deaths and therefore constituted the major determinant of sample size. A survey covering 450,000 population, based on the population structure and death rates from the Tigray Region 2007 census (10), would be expected to include 66,150 children under 5 years of age and record 1,580 under-five deaths over a 1-year period, which should allow reasonably precise estimates of major causes of childhood death. The assumption that under-five deaths would be adequately represented in a sample with sufficient maternal deaths would hold true even allowing for the possibility that fertility and child mortality may have declined substantially since the 2007 census date.

However, since logistics required stratification at a higher than optimal geographical level, a design effect needs to be introduced into the sample size to allow for the cluster-sampling effect as a result of stratification. A design effect of 2 would imply a need to survey a population base of 900,000.

## Sampling process

**Fig. 1:**
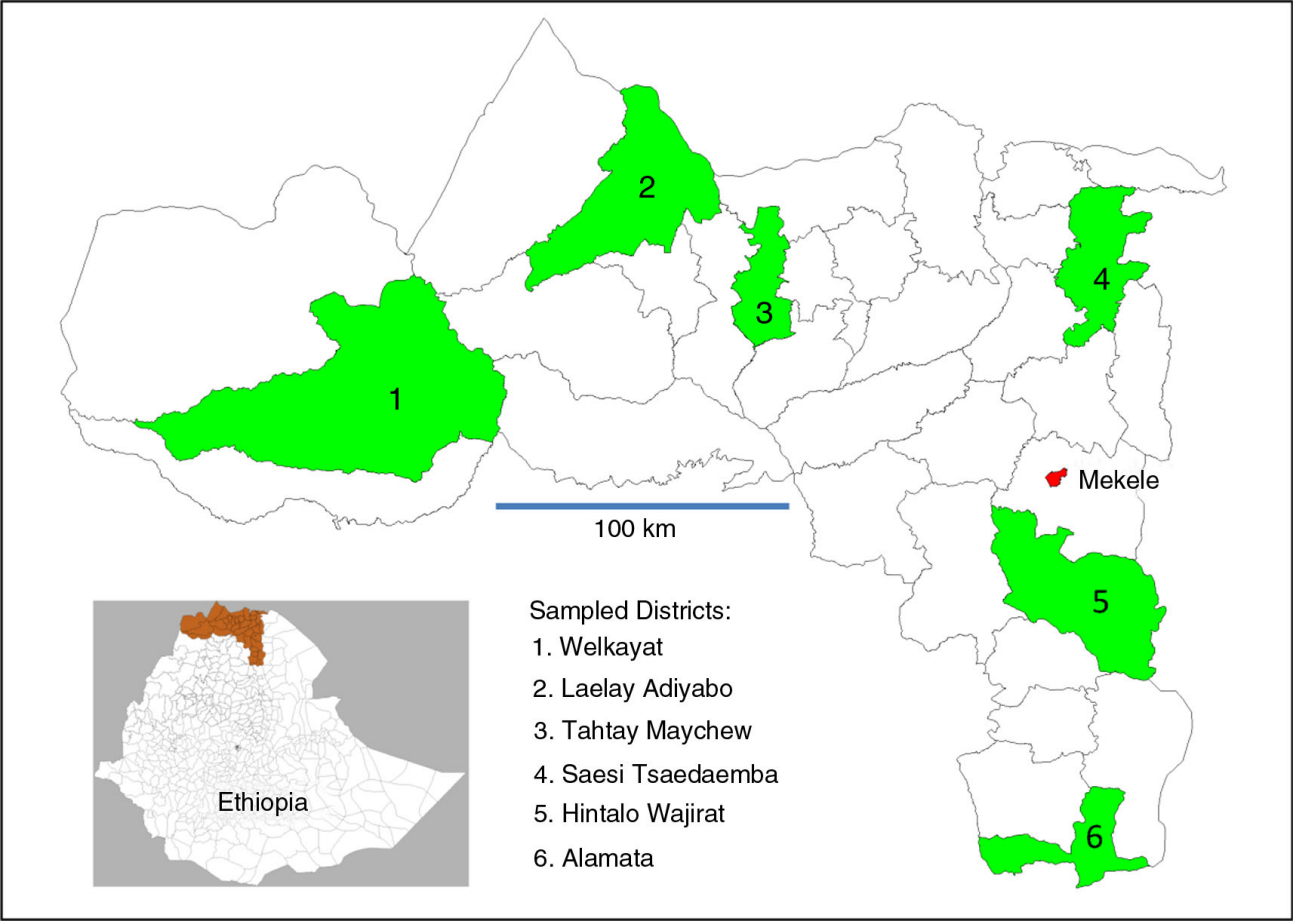
Selected survey districts in Tigray Region, Ethiopia.

With the aim of selecting a geographically representative sample of 900,000 people from Tigray Region's total population of 5.1 million, and since typical district populations are around 150,000 and the region is divided into six zones, the obvious strategy was to randomly select one district from each zone for inclusion in the sample. This was done by assigning a random number (using the Stata 12 *runiform* function) to each district and selecting the district having the highest random number within each zone. As a result of this process, six randomly selected districts were identified as shown in Fig. 1. Despite the statistical disadvantages of the sample being clustered as one district per zone, this approach also brought the advantage of being able to estimate mortality for individual districts (within which considerations of clustering did not apply) with moderate precision. With the same parameter estimates as used above, an MMR of 400 per 100,000 could be estimated at district level as nine deaths over 2,250 births with a 95% confidence interval of 183–758. Using projections from the national 2007 census, the total population of the six selected districts at the time of the survey was estimated at 843,115 living in 183,286 households.

## Survey implementation

The verbal autopsy mortality survey was designed as a two-stage process in each of the six selected districts. The first stage involved visits to every household in the district by health extension workers (15) to identify whether any death to a child under 5 years of age or to a woman aged 15–49 years had occurred in the previous 12-month period. Additionally, the number of live births that had occurred in the household during the same 12-month period was noted.

A total of 337 health extension workers and 34 primary health care unit supervisors (PHCUSs) were trained to undertake this first stage of the survey. They underwent a 1-day training programme covering the purpose of the survey, the information to be collected, the eligible study participants, and the study period. The training was given by two coordinators (one specialised in statistics and one in reproductive health) who had previous experience of verbal autopsy field work. The health extension workers and supervisors were normally resident in the survey areas to which they were assigned, and familiar with the local communities. In Ethiopia, health extension workers’ routine work involves fortnightly visits to all households in their local area, moving around communities on foot, and so they needed no special transportation for this survey. PHCUSs supervised the health extension workers as they undertook this survey on a regular basis. This first stage of the survey involved approximately 2,500 person-days of work.

The second stage involved re-visiting the households where deaths had occurred to undertake verbal autopsy interviews using questionnaires based on the WHO 2012 standard and translated into the local Tigrigna language. For this stage, the 132 health extension workers who had identified deaths in the first stage were involved, together with eight supervisors. They used verbal autopsy log books prepared from the initial survey which included the names, locations, and household identification numbers for the identified deaths. The health extension workers and supervisors were trained for a further 2 days by the same two coordinators and a principal investigator. This training covered methods of undertaking verbal autopsy interviews and in the details of the questionnaires used. One-to-one verbal autopsy role-play exercises were used as part of the training. As usual, the health extension workers travelled around the communities on foot, and the supervisors used vehicles for supervision. The requisite verbal autopsy interviews were successfully completed for 87.5% of child deaths and 98.6% of deaths among women of reproductive age over a period of approximately 20 days, with individual health extension workers completing approximately five verbal autopsies per day. The total work involved was approximately 100 person-days. The major limitation was not the length of the verbal autopsy interviews (approximately 20 min each) but the time taken to move from one household where a death had been identified to the next.

## Survey costs

Although the survey was undertaken by the Tigray Regional Health Bureau using existing staff and resources, and as such it is difficult to put precise costs on every aspect of the work, approximate costs are summarised in Table 1. The total cost of the survey was approximately $60,000.

**Table 1:** Summary of costs (US$) for a cause-specific mortality survey in Tigray Region, Ethiopia

	Salaries	Allowances	Transport	Other	Total
Stage one					
Training	1,157	7,028	513		8,698
Field work	7,381	32,663			40,044
Supervision	1,153	3,024			4,177
Stage two					
Training	488	3,017	513		4,018
Field work	293	1,236			1,529
Supervision	46	1,168			1,214
Other				513	513
Total					60,193

## Discussion

This paper fills an important gap – how to take the available tools for verbal autopsy into an unregistered population in order to obtain reliable estimates of cause-specific mortality at the population level. This particular case-study focuses on Tigray Region in northern Ethiopia, but the general principles would apply to many other places. Tigray Region is mountainous, fairly sparsely populated, with limited infrastructure in rural areas, and so is definitely not an easy option for this kind of work in relation to many other areas.

In this survey, the targets within the population were deaths among women of reproductive age and children under 5 years of age, together with the capture of live births as a denominator. If deaths across the entire population were of interest, the first stage of the field survey would need to capture household population data (number of residents in each age-sex group of interest) to serve as denominator data. This approach was undertaken previously in other parts of Tigray as part of a malaria survey (16).

The overall cost of undertaking this survey can be interpreted in a number of ways. Firstly, it must be remembered that these costs relate to local conditions in Ethiopia and might be different elsewhere. Then, to some, a price-tag of $60,000 to establish how many people had died and of what causes may seem excessive. It might well be argued that these resources would have been better dedicated to curative health services. The alternative view, however, is that this $60,000 survey to determine mortality patterns among a population of 5 million only cost $0.012 per capita. This in turn is an extremely small fraction of the region's per capita expenditure on health and other population-based services. Spending 1 cent per person to get information that is crucial to planning and running an effective health care system seems well justified.

It is important to recognise the important advantages operating here in that the survey process was initiated and implemented from the senior levels of the Tigray Regional Health Bureau. It is not always the case that health service providers and researchers work together as effectively as was evident here. Consequently, we would like to emphasise the extreme importance of having close cooperation and collaboration between local authorities and researchers in order to achieve evidence-based decision making in health care.

In relation to on-going discussions about extending the global scope of civil registration and vital statistics coverage (17), these methodological considerations represent an important stepping stone. There is a general recognition that, for many regions in Africa and Asia, civil registration and vital statistics implementation for the foreseeable future would need to rely on verbal autopsy methods as the only viable approach to cause of death determination. The Sample Vital Registration with Verbal Autopsy (SAVVY) tools developed by MEASURE Evaluation and the US Census Bureau in 2009 (18) set out methods for general population surveillance including verbal autopsy, but there are few papers in the peer-reviewed literature reporting and evaluating their use (19). Furthermore, the SAVVY framework preceded recent developments in automated cause of death coding for verbal autopsy material, and hence was predicated on physician coding. This is not likely to represent a realistic way forward for large-scale vital registration.

The availability of appropriate verbal autopsy methods alone does not provide all the answers in terms of how to implement full civil registration and vital statistics. The questions around how to trace and follow-up all deaths, whether or not they occur at health facilities, are absolutely critical to the process, not least to ensuring the completeness and quality of the mortality data. What is evident from this survey is that tracing the deaths occupied over 95% of the overall effort involved; undertaking the verbal autopsy interviews was a relatively minor part of the overall process. With increasingly available opportunities for automating the verbal autopsy interview and cause of death assignment process, for example, using smart-phone technology (9), it becomes very clear that logistic issues remain as the major challenge preventing reliable and systematic identification of deaths in populations. What is clear from the experiences reported here, however, is that it is possible to undertake these tasks using interviewers with relatively basic training, but who know and understand the communities involved, such as the health extension workers in Ethiopia.

For civil registration and vital statistics purposes, in which registration of deaths and verbal autopsies should preferably become a continuous, routine process, it would probably be more appropriate to think of strategies involving key informants, community leaders, etc. as part of routine longitudinal systems of identifying deaths. Nevertheless, as part of a transitional stage from not registering deaths at all, but before complete civil registration is successfully implemented, there may well be a need for intermediate strategies such as the population survey approach outlined here.
